# Axisymmetric Fractional Diffusion with Mass Absorption in a Circle under Time-Harmonic Impact

**DOI:** 10.3390/e24071002

**Published:** 2022-07-20

**Authors:** Yuriy Povstenko, Tamara Kyrylych

**Affiliations:** Department of Mathematics and Computer Sciences, Faculty of Science and Technology, Jan Dlugosz University in Czestochowa, al. Armii Krajowej 13/15, 42-200 Czestochowa, Poland; t.kyrylych@ujd.edu.pl

**Keywords:** fractional calculus, Caputo derivative, Mittag-Leffler function, time-harmonic impact, quasi-steady state, finite Hankel transform

## Abstract

The axisymmetric time-fractional diffusion equation with mass absorption is studied in a circle under the time-harmonic Dirichlet boundary condition. The Caputo derivative of the order 0<α≤2 is used. The investigated equation can be considered as the time-fractional generalization of the bioheat equation and the Klein–Gordon equation. Different formulations of the problem for integer values of the time-derivatives α=1 and α=2 are also discussed. The integral transform technique is employed. The outcomes of numerical calculations are illustrated graphically for different values of the parameters.

## 1. Introduction

Physical processes take place at various structural levels. For this reason, it is necessary to use specific physical concepts and mathematical tools corresponding to every structural level, combining the descriptions of processes at different structural levels. One of these physical concepts is that of memory effects, for which the corresponding mathematical tool is fractional calculus. The theory of integrals and derivatives of non-integer order has numerous applications in different fields of study (see, for example, [1,2,3,4,5,6,7,8,9,10,11], among many others). The interested reader is also referred to a recent review article [12] containing an extensive bibliography.

Classical heat conduction is based on the Fourier law [13], and classical diffusion is based on the Fick law [14]. Both laws can be written as

(1) q=−kgradu,

where q is the corresponding flux, *u* denotes temperature or mass concentration, and *k* is the thermal (diffusion) conductivity. In combination with the associated balance equation, the constitutive Equation (1) leads to the heat conduction (diffusion) equation

(2) $$\frac{\partial u}{\partial t}=a\Delta u,$$

where *a* is the diffusivity coefficient. For heat conduction, a=k/(ρC), where *C* denotes the specific heat capacity and ρ is the mass density. For diffusion, a=k/ρ.

The classical Fourier law and the Fick law (1), together with the standard heat conduction (diffusion) Equation (2), are quite sufficient for describing many physical processes. However, in bodies with complex internal structures, where physical processes occur at different structural levels, the standard parabolic equations are inadequate to take into account the characteristic features of transport processes in such media.

In non-classical theories, the Fourier law, the Fick law, and the associated parabolic heat conduction (diffusion) equation are replaced by more general equations. It should be noted that the constitutive equations that describe memory effects have considerable promise in this area [15,16,17,18].

The time-non-local dependences between the flux and the temperature (concentration) gradient with a “long-tailed” power kernel [7,8,19,20,21] can be formulated in terms of the Riemann–Liouville fractional integrals and derivatives

(3) $$\begin{array}{ccc} q(t) & \;\;\;= & \;\;\;-kD_{RL}^{1-\alpha }\mathrm{grad}\;u\left(t\right),\;0<\alpha \leq 1, \end{array}$$

(4) $$\begin{array}{ccc} q(t) & \;\;\;= & \;\;\;-kI^{\alpha -1}\mathrm{grad}\;u\left(t\right),\;\;1<\alpha \leq 2, \end{array}$$

and in combination with the appropriate balance equation yield the time-fractional diffusion-wave equation with the Caputo fractional derivative

(5) $$\frac{\partial^{\alpha }u}{\partial t^{\alpha }}=a\Delta u,\;0<\alpha \leq 2.$$

The definitions of fractional integrals and derivatives and their Laplace transform rules can be found, for example, in [1,2].

In fractional calculus, there is no definitive line between differentiation and integration. For this reason, some authors (see, for example, [1]) do not use a separate notation for the Riemann–Liouville fractional integral, assuming that $D_{RL}^{-\beta }u\left(t\right)$ with β>0 denotes a Riemann–Liouville integral of fractional order. With such a notation, the constitutive Equations (3) and (4), can be written as

(6) $$q\left(t\right)=-kD_{RL}^{1-\alpha }\mathrm{grad}\;u\left(t\right),\;0<\alpha \leq 2.$$

Details on obtaining the time-fractional diffusion-wave Equation (5) from the mass balance equation and the constitutive Equation (6) for the mass flux can be found in [21].

The diffusion-wave Equation (5) describes many important physical phenomena in different media, and in the case 1<α<2 interpolates between the diffusion Equation (2) for α=1 and the wave equation

(7) $$\frac{\partial^{2}u}{\partial t^{2}}=a\Delta u$$

for α=2, governing so-called “ballistic diffusion”. It should be noted that Equation (7) was evolved in the theory of heat and mass transport as a consequence of the generalized time-non-local constitutive equation for the corresponding flux with “full memory” [18,22].

In a medium with heat absorption or for an irreversible chemical reaction of the first order, an additional linear source term appears in Equation (2) [23,24]:

(8) $$\frac{\partial u}{\partial t}=a\Delta u-bu.$$

Similar equations describe heat conduction in solids that lose heat by radiation to a surrounding medium [25,26] and also arise in the theory of bioheat transfer [27,28].

The Klein–Gordon equation

(9) $$\frac{\partial^{2}u}{\partial t^{2}}=a\Delta u-bu$$

is of great importance in solid state physics, nonlinear optics, and quantum field theory [29,30]. The corresponding counterpart of Equation (5)

(10) $$\frac{\partial^{\alpha }u}{\partial t^{\alpha }}=a\Delta u-bu,\;0<\alpha \leq 2,$$

contains the parabolic Equation (8) and the hyperbolic Equation (9) as particular cases.

Ångström’s research [31] was the first in which the heat conduction equation was investigated under time-harmonic conditions. An extensive literature on “oscillatory diffusion” can be found in [32,33]. In this context, both the time-harmonic source term and the time-harmonic boundary conditions were considered.

In many papers devoted to this subject, the so-called “quasi-steady-state”solutions were studied (see, e.g., [25,34,35,36]). In this case, the solution of the classical diffusion Equation (2) or the equation with mass absorption (8) is sought for as a product of a function of spatial coordinates and the harmonic term

(11) $$u\left(x,t\right)=U\left(x\right)e^{i\omega t}.$$

For the time-fractional diffusion Equations (5) or (10), such an approach is impossible because, for the fractional Caputo derivative of the exponential function, we have [37]

(12) $$\frac{d^{\alpha }e^{\lambda t}}{dt^{\alpha }}=\lambda^{\alpha }e^{\lambda t}\;\frac{\gamma \left(n-\alpha ,\lambda t\right)}{\Gamma \left(n-\alpha \right)}\neq \lambda^{\alpha }e^{\lambda t},\;n-1<\alpha <n,$$

where Γ(x) is the gamma function and γ(a,x) is the incomplete gamma function

(13) $$\gamma \left(a,x\right)=\int_{0}^{x}e^{-t}t^{a-1}dt.$$

In [38], the time-fractional diffusion equation with mass absorption and a source term varying harmonically in time was considered in the domain −∞<x<∞. In [39], this equation was studied for a sphere. In the present paper, we investigate the axisymmetric time-fractional diffusion equation with mass absorption (10) in a circle, under the time-harmonic Dirichlet boundary condition. The Laplace transform with respect to time and the finite Hankel transform with respect to the radial coordinate are employed. For integer values of the order of the time-derivative (see Equations (8) and (9)), the quasi-steady-state solutions are also discussed. The outcomes of numerical simulations are illustrated graphically for different values of the parameters.

## 2. Integer Order of Time-Derivative

### 2.1. Diffusion Equation with Mass Absorption (α=1)

We start our analysis from the quasi-steady-state solution of the axisymmetric diffusion equation with mass absorption in a circle 0<r<R

(14) $$\frac{\partial u}{\partial t}=a\left(\frac{\partial^{2}u}{\partial r^{2}}+\frac{1}{r}\;\frac{\partial u}{\partial r}\right)-bu,$$

under time-harmonic conditions at the boundary r=R of a circle

(15) $$r=R:\;\;u=u_{0}\;e^{i\omega t}.$$

Assuming that

(16) $$u\left(r,t\right)=U\left(r\right)e^{i\omega t},$$

we obtain

(17) $$\frac{d^{2}U}{dr^{2}}+\frac{1}{r}\;\frac{dU}{dr}-\frac{b+i\omega }{a}\;U=0,$$

(18) $$r=R:\;\;U=u_{0}.$$

The general solution of Equation (17) is expressed in terms of the modified Bessel functions

(19) $$U\left(r\right)=C_{1}I_{0}\left[r\sqrt{(b+i\omega )/a}\right]+C_{2}K_{0}\left[r\sqrt{(b+i\omega )/a}\right].$$

The boundedness condition at the origin r=0 implies that $C_{2}=0$, and the boundary condition (18) gives

(20) $$U\left(r\right)=u_{0}\;\frac{I_{0}\left[r\sqrt{(b+i\omega )/a}\right]}{I_{0}\left[R\sqrt{(b+i\omega )/a}\right]}.$$

It should be noted that Pennes [27] considered the axisymmetric bioheat equation in a cylindrical domain 0<r<R (as a model of the forearm) and obtained the solution (20) in the case ω=0 (see also [28]).

Hence, the quasi-steady-state solution reads

(21) $$u\left(r,t\right)=u_{0}\;\frac{I_{0}\left[r\sqrt{(b+i\omega )/a}\right]}{I_{0}\left[R\sqrt{(b+i\omega )/a}\right]}\;e^{i\omega t}.$$

**Figure 1:**
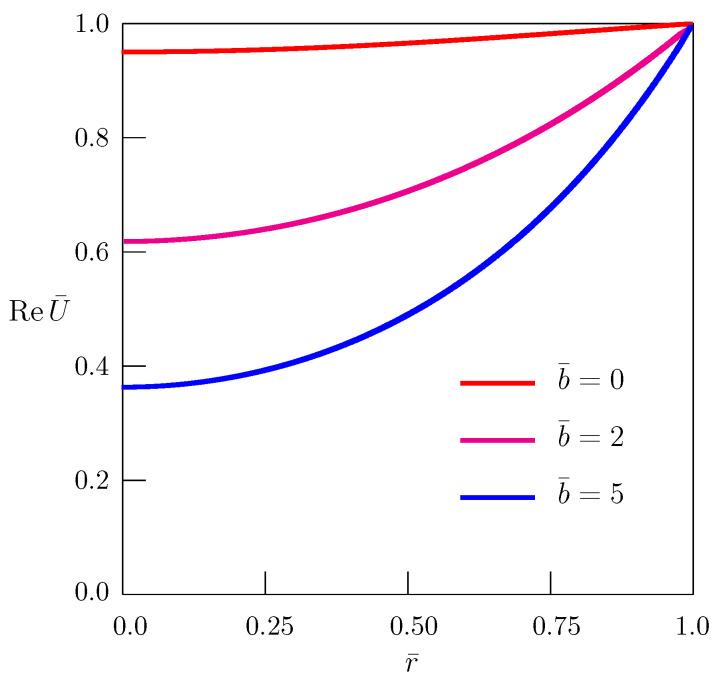
Dependence of the real part of the amplitude of the quasi-steady-state solution to the diffusion equation (α=1) on the radial coordinate $\bar{r}$ for different values of the mass absorption parameter $\bar{b}$; $\;\bar{\omega }=\pi /3$.

The dependence of the real part of the amplitude U(r) (20) of the quasi-steady-state solution (21) is shown in Figure 1 for different values of the mass absorption parameter *b*. The following nondimensional quantities

(22) $$\bar{U}=\frac{U}{u_{0}},\;\bar{r}=\frac{r}{R},\;\bar{t}=\frac{a}{R^{2}}\;t,\;\bar{\omega }=\frac{R^{2}}{a}\;\omega ,\;\bar{b}=\frac{R^{2}}{a}\;b$$

were introduced.

Next, we investigate Equation (14) under the boundary condition (15) without the assumption (16) for the zero initial condition

(23) t=0:u(r,t)=0.

The finite Hankel transform with respect to the radial coordinate *r* (see Appendix A) and the Laplace transform with respect to time *t* give the solution in the transform domain

(24) $${\hat{u}}^{*}\left(\xi_{k},s\right)=aRu_{0}\;\xi_{k}J_{1}\left(R\xi_{k}\right)\;\frac{1}{s-i\omega }\;\frac{1}{s+a\xi_{k}^{2}+b}.$$

Inversion of the integral transform using Equation (A2) from Appendix A and Equation (A5) from Appendix B results in the solution

(25) $$u\left(r,t\right)=\frac{2au_{0}}{R}\;\sum_{k=1}^{\infty }\frac{\xi_{k}J_{0}\left(r\xi_{k}\right)}{J_{1}\left(R\xi_{k}\right)\left(a\xi_{k}^{2}+b+i\omega \right)}\left[e^{i\omega t}-e^{-\left(a\xi_{k}^{2}+b\right)t}\right].$$

Taking into account the following series containing zeros of the Bessel function $J_{0}\left(r\right)$ [40]

(26) $$\sum_{k=1}^{\infty }\frac{R\xi_{k}J_{0}\left(r\xi_{k}\right)}{J_{1}\left(R\xi_{k}\right)\;\left(R^{2}\xi_{k}^{2}-c^{2}\right)}=\frac{1}{2}\;\frac{J_{0}\left(cr/R\right)}{J_{0}\left(c\right)},$$

we arrive at

(27) $$\begin{array}{ccc} u(r,t) & \;\;\;= & \;\;\;u_{0}\;\frac{I_{0}\left[r\sqrt{(b+i\omega )/a}\right]}{I_{0}\left[R\sqrt{(b+i\omega )/a}\right]}\;e^{i\omega t}\\ & \;\;\;- & \;\;\;\frac{2au_{0}}{R}\;\sum_{k=1}^{\infty }\frac{\xi_{k}J_{0}\left(r\xi_{k}\right)}{J_{1}\left(R\xi_{k}\right)\left[a\xi_{k}^{2}+b+i\omega \right]}e^{-\left(a\xi_{k}^{2}+b\right)t}. \end{array}$$

The first term in Equation (27) coincides with the quasi-steady-state solution (21), and the second term describes the transient process.

### 2.2. Klein–Gordon Equation (α=2)

The quasi-steady-state solution of the Klein–Gordon equation

(28) $$\frac{\partial^{2}u}{\partial t^{2}}=a\left(\frac{\partial^{2}u}{\partial r^{2}}+\frac{1}{r}\;\frac{\partial u}{\partial r}\right)-bu,\;0<r<R,$$

under the time-harmonic Dirichlet boundary condition

(29) $$r=R:\;\;u=u_{0}\;e^{i\omega t}$$

is obtained under assumption (16). In this case, the equation for the amplitude function U(r) takes the form

(30) $$\frac{d^{2}U}{dr^{2}}+\frac{1}{r}\;\frac{dU}{dr}-\frac{b-\omega^{2}}{a}\;U=0.$$

Depending on the sign of the coefficient $b-\omega^{2}$, the general solution of Equation (30) is expressed in terms of the Bessel functions $J_{0}\left[r\sqrt{\left(\omega^{2}-b\right)/a}\right]$ and $Y_{0}\left[r\sqrt{\left(\omega^{2}-b\right)/a}\right]$ for $\omega^{2}>b$ and the modified Bessel functions $I_{0}\left[r\sqrt{\left(b-\omega^{2}\right)/a}\right]$ and $K_{0}\left[r\sqrt{\left(b-\omega^{2}\right)/a}\right]$ for $b>\omega^{2}$. Under the assumption of boundedness at the origin r=0, accounting for the boundary condition (29) leads to the expression for the amplitude

(31) $$U\left(r\right)=\left\{\begin{array}{cc} \;\;u_{0}\;\frac{J_{0}\left[r\sqrt{\left(\omega^{2}-b\right)/a}\right]}{J_{0}\left[R\sqrt{\left(\omega^{2}-b\right)/a}\right]}\;, & \;\;\omega^{2}>b,\\ \;\;u_{0}\;\frac{I_{0}\left[r\sqrt{\left(b-\omega^{2}\right)/a}\right]}{I_{0}\left[R\sqrt{\left(b-\omega^{2}\right)/a}\right]}\;, & \;\;b>\omega^{2}, \end{array}\right.$$

and to the corresponding quasi-steady-state solution

(32) $$u\left(r,t\right)=\left\{\begin{array}{cc} \;\;u_{0}\;\frac{J_{0}\left[r\sqrt{\left(\omega^{2}-b\right)/a}\right]}{J_{0}\left[R\sqrt{\left(\omega^{2}-b\right)/a}\right]}\;e^{i\omega t}, & \;\;\omega^{2}>b,\\ \;\;u_{0}\;\frac{I_{0}\left[r\sqrt{\left(b-\omega^{2}\right)/a}\right]}{I_{0}\left[R\sqrt{\left(b-\omega^{2}\right)/a}\right]}\;e^{i\omega t}, & \;\;b>\omega^{2}. \end{array}\right.$$

**Figure 2:**
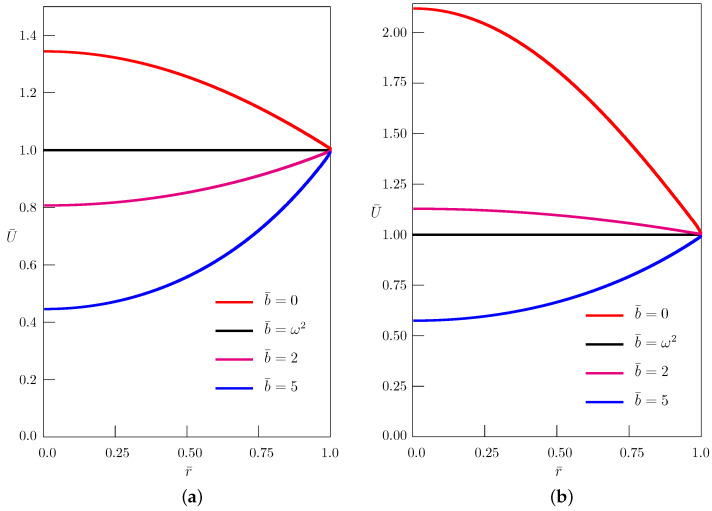
Dependence of the amplitude of the quasi-steady-state solution to the Klein–Gordon equation (α=2) on the radial coordinate $\bar{r}$ for different values of the mass absorption parameter $\bar{b}$: (**a**) $\bar{\omega }=\pi /3$; (**b**) $\bar{\omega }=\pi /2$.

The dependence of the amplitude U(r) (31) of the quasi-steady-state solution (32) is shown in Figure 2 for different interrelations between the mass absorption parameter *b* and the angular frequency ω.

The nondimensional quantities were chosen as

(33) $$\bar{U}=\frac{U}{u_{0}},\;\bar{r}=\frac{r}{R},\;\bar{t}=\frac{\sqrt{a}}{R}\;t,\;\bar{\omega }=\frac{R}{\sqrt{a}}\;\omega ,\;\bar{b}=\frac{R^{2}}{a}\;b.$$

Now, we investigate the transition regime considering the Klein–Gordon Equation (28) under the harmonic boundary condition (29) and zero initial conditions

(34) $$\begin{array}{ccc} t=0: & & u=0, \end{array}$$

(35) $$\begin{array}{ccc} t=0: & & \frac{\partial u}{\partial t}=0. \end{array}$$

The integral transform technique results in the solution in the transform domain

(36) $${\hat{u}}^{*}\left(\xi_{k},s\right)=aRu_{0}\;\xi_{k}J_{1}\left(R\xi_{k}\right)\;\frac{1}{s-i\omega }\;\frac{1}{s^{2}+a\xi_{k}^{2}+b}.$$

Equations (A2) from Appendix A and (A6) from Appendix B allow us to obtain the sought-for solution

(37) $$\begin{array}{ccc} u(r,t) & \;= & \;\frac{2au_{0}}{R}\sum_{k=1}^{\infty }\frac{\xi_{k}J_{0}\left(r\xi_{k}\right)}{J_{1}\left(R\xi_{k}\right)\left(a\xi_{k}^{2}+b-\omega^{2}\right)}\\ & \;\times & \;\left[e^{i\omega t}-\cos\left(\sqrt{a\xi_{k}^{2}+b}\;t\right)-\frac{i\omega }{\sqrt{a\xi_{k}^{2}+b}}\sin\left(\sqrt{a\xi_{k}^{2}+b}\;t\right)\right]. \end{array}$$

Taking into account Equation (26), we finally obtain

(38) $$u\left(r,t\right)=\left\{\begin{array}{cc} \;\;u_{0}\;\frac{J_{0}\left[r\sqrt{\left(\omega^{2}-b\right)/a}\right]}{J_{0}\left[R\sqrt{\left(\omega^{2}-b\right)/a}\right]}\;e^{i\omega t}-T\left(r,t\right), & \;\;\omega^{2}>b,\\ \;\;u_{0}\;\frac{I_{0}\left[r\sqrt{\left(b-\omega^{2}\right)/a}\right]}{I_{0}\left[R\sqrt{\left(b-\omega^{2}\right)/a}\right]}\;e^{i\omega t}-T\left(r,t\right), & \;\;b>\omega^{2}, \end{array}\right.$$

where

(39) $$\begin{array}{ccc} T(r,t) & \;\;= & \;\;\frac{2au_{0}}{R}\sum_{k=1}^{\infty }\frac{\xi_{k}J_{0}\left(r\xi_{k}\right)}{J_{1}\left(R\xi_{k}\right)\left(a\xi_{k}^{2}+b-\omega^{2}\right)}\\ & \;\;\times & \;\;\;\left[\cos\left(\sqrt{a\xi_{k}^{2}+b}\;t\right)+\frac{i\omega }{\sqrt{a\xi_{k}^{2}+b}}\sin\left(\sqrt{a\xi_{k}^{2}+b}\;t\right)\right]. \end{array}$$

It is evident that the first terms in (38) coincide with the quasi-steady-state solution (32), whereas the term T(r,t) describes the transient regime.

## 3. Time-Fractional Diffusion-Wave Equation

Consider the time-fractional diffusion-wave equation with mass absorption in a circle

(40) $$\frac{\partial^{\alpha }u}{\partial t^{\alpha }}=a\left(\frac{\partial^{2}u}{\partial r^{2}}+\frac{1}{r}\;\frac{\partial u}{\partial r}\right)-bu,\;0<r<R,\;\;0<\alpha \leq 2,$$

under zero initial conditions

(41) $$\begin{array}{ccc} t=0: & & u=0,\;0<\alpha \leq 2, \end{array}$$

(42) $$\begin{array}{ccc} t=0: & & \frac{\partial u}{\partial t}=0,\;\;1<\alpha \leq 2, \end{array}$$

and the time-harmonic Dirichlet boundary condition

(43) $$r=R:\;\;u=u_{0}\;e^{i\omega t}.$$

We also assume the boundedness condition at the origin:

(44) r=0:u≠∞.

The finite Hankel transform with respect to the radial coordinate *r* and the Laplace transform with respect to time *t* give the solution in the transform domain

(45) $${\hat{u}}^{*}\left(\xi_{k},s\right)=aRu_{0}\;\xi_{k}J_{1}\left(R\xi_{k}\right)\;\frac{1}{s-i\omega }\;\frac{1}{s^{\alpha }+a\xi_{k}^{2}+b}.$$

It is convenient to rearrange Equation (45) as

(46) $${\hat{u}}^{*}\left(\xi_{k},s\right)=\frac{aRu_{0}\;\xi_{k}J_{1}\left(R\xi_{k}\right)}{a\xi_{k}^{2}+b}\left[\frac{1}{s-i\omega }-\frac{s^{\alpha -1}}{s^{\alpha }+a\xi_{k}^{2}+b}-\frac{i\omega }{s-i\omega }\;\frac{s^{\alpha -1}}{s^{\alpha }+a\xi_{k}^{2}+b}\right].$$

The inverse transforms taking into account the convolution theorem allow us to obtain the solution

(47) $$\begin{array}{ccc} & & \;\;\;\;\;\;\;\;\;\;\;\;\;\;\;\;\;\;\;\;\;\;u\left(r,t\right)=\frac{2au_{0}}{R}\;\sum_{k=1}^{\infty }\frac{\xi_{k}J_{0}\left(r\xi_{k}\right)}{J_{1}\left(R\xi_{k}\right)\left(a\xi_{k}^{2}+b\right)}\\ & & \;\;\;\;\;\;\;\;\;\;\;\;\;\;\;\;\;\;\;\;\;\;\times \left\{e^{i\omega t}-E_{\alpha }\left[-\left(a\xi_{k}^{2}+b\right)t^{\alpha }\right]-i\omega \int_{0}^{t}e^{i\omega (t-\tau )}\;E_{\alpha }\left[-\left(a\xi_{k}^{2}+b\right)\tau^{\alpha }\right]d\tau \right\}, \end{array}$$

where Equation (A7) from Appendix B has been used for the inverse Laplace transform, with $E_{\alpha }\left(z\right)$ as the Mittag-Leffler function.

Using the relation (26), we rewrite Equation (47) in the following form

(48) $$\begin{array}{ccc} & & u\left(r,t\right)=u_{0}\;\frac{I_{0}\left(r\sqrt{b/a}\right)}{I_{0}\left(R\sqrt{b/a}\right)}\;e^{i\omega t}-\frac{2au_{0}}{R}\;\sum_{k=1}^{\infty }\frac{\xi_{k}J_{0}\left(r\xi_{k}\right)}{J_{1}\left(R\xi_{k}\right)\left(a\xi_{k}^{2}+b\right)}\\ & & \times \left\{E_{\alpha }\left[-\left(a\xi_{k}^{2}+b\right)t^{\alpha }\right]+i\omega \int_{0}^{t}e^{i\omega (t-\tau )}\;E_{\alpha }\left[-\left(a\xi_{k}^{2}+b\right)\tau^{\alpha }\right]d\tau \right\}. \end{array}$$

Due to the rearrangement (46), the first term in the solution (48) satisfies the boundary condition (43); other terms at the boundary r=R are equal to zero (see Equation (A3) in Appendix A).

The particular case of Equation (48) corresponding to b=0 and ω=0

(49) $$u\left(r,t\right)=u_{0}\left[1-\frac{2}{R}\;\sum_{k=1}^{\infty }\frac{J_{0}\left(r\xi_{k}\right)}{\xi_{k}J_{1}\left(R\xi_{k}\right)}E_{\alpha }\left(-a\xi_{k}^{2}t^{\alpha }\right)\right]$$

was obtained in [41].

It is worthwhile to analyze the particular cases of the solution (48) corresponding to integer values of the order of time-derivatives α=1 and α=2. Since $E_{1}\left(-x\right)=e^{-x}$, the integral term in (48) gives

(50) $$\frac{i\omega }{a\xi_{k}^{2}+b+i\omega }\left[e^{i\omega t}-e^{-\left(a\xi_{k}^{2}+b\right)t}\right].$$

Using partial fraction decomposition

(51) $$\frac{i\omega }{\left(a\xi_{k}^{2}+b\right)\left(a\xi_{k}^{2}+b+i\omega \right)}=\frac{1}{a\xi_{k}^{2}+b}-\frac{1}{a\xi_{k}^{2}+b+i\omega }$$

and the series representation (26), after simple mathematical treatment we arrive at the solution (27).

Similarly, $E_{2}\left(-x\right)=\cos\left(\sqrt{x}\right)$, and the integral term in (48) reduces to

(52) $$\frac{1}{a\xi_{k}^{2}+b-\omega^{2}}\left[-\omega^{2}e^{i\omega t}+\omega^{2}\cos\left(\sqrt{a\xi_{k}^{2}+b}t\right)+i\omega \sqrt{a\xi_{k}^{2}+b}\;\sin\left(\sqrt{a\xi_{k}^{2}+b}t\right)\right].$$

The partial fraction decomposition

(53) $$\frac{\omega^{2}}{\left(a\xi_{k}^{2}+b\right)\left(a\xi_{k}^{2}+b-\omega^{2}\right)}=\frac{1}{a\xi_{k}^{2}+b-\omega^{2}}-\frac{1}{a\xi_{k}^{2}+b}$$

taking into account (26) converts (48) to the solution (38)–(39).

**Figure 3:**
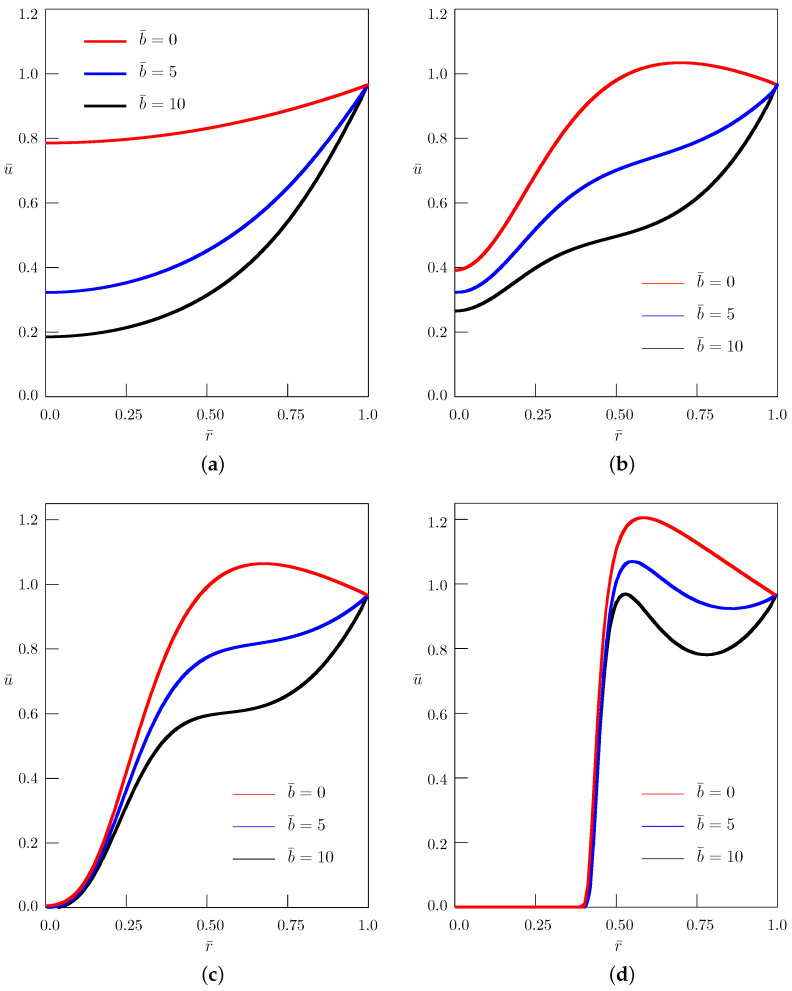
Dependence of the solution on the radial coordinate $\bar{r}$ for different values of the mass absorption parameter $\bar{b}$; $\bar{t}=0.5$, $\bar{\omega }=\pi /6$: (**a**) α=0.5; (**b**) α=1.65; (**c**) α=1.75; (**d**) α=1.95.

**Figure 4:**
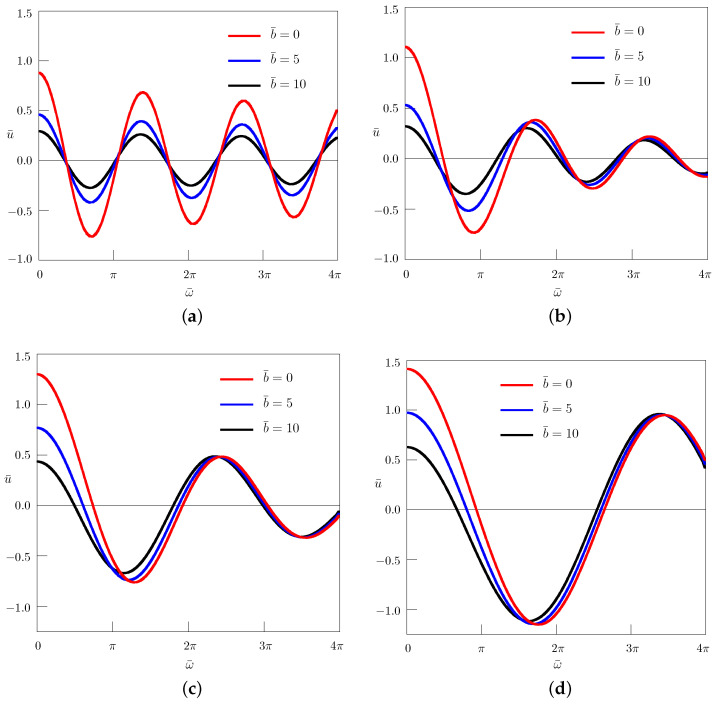
Dependence of the solution on the angular frequency $\bar{\omega }$ for different values of the mass absorption parameter $\bar{b}$; $\bar{t}=0.5$, $\bar{r}=0.75$: (**a**) α=0.5; (**b**) α=1.25; (**c**) α=1.75; (**d**) α=1.95.

The solution (48) is presented in Figure 3 and Figure 4 for different values of the nondimensional parameters:

(54) $$\bar{u}=\frac{u}{u_{0}},\;\bar{r}=\frac{r}{R},\;\bar{t}=\frac{a^{1/\alpha }}{R^{2/\alpha }}\;t,\;\bar{\omega }=\frac{R^{2/\alpha }}{a^{1/\alpha }}\;\omega ,\;\bar{b}=\frac{R^{2}}{a}\;b.$$

## 4. Concluding Remarks

We considered the axisymmetric diffusion equation with mass absorption and with the Caputo time-derivative of the order 0<α≤2 in a circle, under the Dirichlet boundary condition varying harmonically in time. For integer values of the order of the time-derivative, both the quasi-steady-state solutions and the transient processes were studied. For 1<α<2, the obtained solution interpolates between the solutions to the bioheat equation and the Klein–Gordon equation. When α=2, the corresponding solution contains the wave front which is approximated by the obtained solution when α approaches 2 (see Figure 3d, where the wave front is located at $\bar{r}=0.5$). We restricted ourselves to positive values of the mass absorption parameter *b*. It is evident from the figures that with increasing *b*, the amplitude of oscillations decreases. Figure 4 shows that in the case 1<α<2, for sufficiently large values of the angular frequency $\bar{\omega }$, the influence of the mass absorption parameter becomes negligible. The solutions are obtained in the form of a series involving zeros of the Bessel function $J_{0}\left(r\right)$, which are tabulated in [42,43]. The large numbers of zeros can be calculated according to the McMahon asymptotic expansion [43]. The Mittag-Leffler function $E_{\alpha }\left(-x\right)$ appearing in the solutions was evaluated using the algorithms proposed in [44] (see also [45]). Similarly to the standard bioheat transfer equation, the solutions to the fractional bioheat equation could have medical applications.

## Data Availability

Not applicable.

## Appendix A

For the Dirichlet boundary condition at the boundary r=R of a circle, the finite Hankel transform of the zeroth order is defined as [8,46]

(A1) $$H\left\{f\left(r\right)\right\}=\hat{f}\left(\xi_{k}\right)=\int_{0}^{R}f\left(r\right)\;J_{0}\left(r\xi_{k}\right)\;r\;dr,$$

(A2) $$H^{-1}\left\{\hat{f}\left(\xi_{k}\right)\right\}=f\left(r\right)=\frac{2}{R^{2}}\sum_{k=1}^{\infty }\hat{f}\left(\xi_{k}\right)\frac{J_{0}\left(r\xi_{k}\right)}{[J_{1}{\left(R\xi_{k}\right]}^{2}},$$

where the hat denotes the transform, $J_{n}\left(r\right)$ is the Bessel function of the first kind of order *n*, and the sum is over all the positive roots of the zeroth-order Bessel function

(A3) $$J_{0}\left(R\xi_{k}\right)=0.$$

In this case

(A4) $$H\left\{\frac{d^{2}f\left(r\right)}{dr^{2}}+\frac{1}{r}\frac{df(r)}{dr}\right\}=-\xi_{k}^{2}\hat{f}\left(\xi_{k}\right)+R\xi_{k}J_{1}\left(R\xi_{k}\right)f\left(R\right).$$

## Appendix B

The formulaes (A5) and (A6) for the inverse Laplace transform are taken from [47,48]:

(A5) $$L^{-1}\left\{\frac{1}{\left(s-p)(s-q\right)}\right\}=\frac{1}{p-q}\left(e^{pt}-e^{qt}\right),$$

(A6) $$L^{-1}\left\{\frac{1}{\left(s+p\right)\left(s^{2}+q^{2}\right)}\right\}=\frac{1}{p^{2}+q^{2}}\left[e^{-pt}-\cos\left(qt\right)+\frac{p}{q}\;\sin\left(qt\right)\right].$$

The Mittag-Leffler function $E_{\alpha }\left(z\right)$ plays an essential role in fractional calculus due to the formula [1,2]

(A7) $$L^{-1}\left\{\frac{s^{\alpha -1}}{s^{\alpha }+q}\right\}=E_{\alpha }\left(-qt^{\alpha }\right),$$

where

(A8) $$E_{\alpha }\left(z\right)=\sum_{k=0}^{\infty }\frac{z^{k}}{\Gamma (\alpha k+1)},\;\;\;\alpha >0,\;\;z\in C.$$
